# Protective Role of Histidine Supplementation Against Oxidative Stress Damage in the Management of Anemia of Chronic Kidney Disease

**DOI:** 10.3390/ph11040111

**Published:** 2018-10-21

**Authors:** Mayra Vera-Aviles, Eleni Vantana, Emmy Kardinasari, Ngat L. Koh, Gladys O. Latunde-Dada

**Affiliations:** King’s College London, Department of Nutritional Sciences, Faculty of Life Sciences and Medicine, Franklin-Wilkins Building, 150 Stamford Street, London SE1 9NH, UK; mayra.vera_aviles@kcl.ac.uk (M.V.-A.); el.vantana@outlook.com (E.V.); ekardinasari@gmail.com (E.K.); lea.koh@gmail.com (N.L.K.)

**Keywords:** histidine, iron, anemia, oxidative stress, kidney

## Abstract

Anemia is a major health condition associated with chronic kidney disease (CKD). A key underlying cause of this disorder is iron deficiency. Although intravenous iron treatment can be beneficial in correcting CKD-associated anemia, surplus iron can be detrimental and cause complications. Excessive generation of reactive oxygen species (ROS), particularly by mitochondria, leads to tissue oxidation and damage to DNA, proteins, and lipids. Oxidative stress increase in CKD has been further implicated in the pathogenesis of vascular calcification. Iron supplementation leads to the availability of excess free iron that is toxic and generates ROS that is linked, in turn, to inflammation, endothelial dysfunction, and cardiovascular disease. Histidine is indispensable to uremic patients because of the tendency toward negative plasma histidine levels. Histidine-deficient diets predispose healthy subjects to anemia and accentuate anemia in chronic uremic patients. Histidine is essential in globin synthesis and erythropoiesis and has also been implicated in the enhancement of iron absorption from human diets. Studies have found that L-histidine exhibits antioxidant capabilities, such as scavenging free radicals and chelating divalent metal ions, hence the advocacy for its use in improving oxidative stress in CKD. The current review advances and discusses evidence for iron-induced toxicity in CKD and the mechanisms by which histidine exerts cytoprotective functions.

## 1. Introduction

Chronic kidney disease (CKD) is a generic term that includes the majority of renal disorders. Anemia, an invariable consequence of CKD, is higher in patients with renal disease compared to the unaffected population (15.4% vs. 7.6%, respectively) globally according to the 2014 outcome of the National Health and Nutrition Examination Survey (NHANES) [1]. Judging by the glomerular filtrate rate, CKD is classified in stages from 1–5, with 5 being the last stage, also known as end-stage renal disease (ESRD) [2]. In the U.K., the prevalence of stages 3–5 CKD is estimated to be 9% of the adult population [3]. Approximately 50% of patients with CKD in the U.S. are reported to be anemic [4]. Observational studies also reported a 13% increased risk of hospitalizations for patients with low hematocrits [5,6], as well as 6% increased risk of cardiovascular events per 10 g/L decrease in hemoglobin (Hb), for patients with anemia in CKD [7]. Data from patients with hemodialysis from five European countries showed that lower hemoglobin levels are associated with increased morbidity and mortality [8]. This result is of particular public health concern as anemia in CKD has been reported to significantly reduce quality of life compared to the general population, with Hb levels as the predictive factor [9]. Oxidative stress and chronic inflammation are hallmarks of CKD; the magnitude of the resultant adverse consequences ranges across the different stages of the manifestation of the disorder and depends on the nature of the therapy employed. The origin of oxidative stress in CKD is varied and includes toxicity induced by excess iron supplements, uremic toxins, and the burden imposed by the hemodialysis process and the equipment employed. Consequently, the inflammation that ensues is associated with elevated ferritin and hepcidin levels; the latter inhibits ferroportin, which blocks iron efflux into circulation. This results in low iron availability for erythropoiesis and hyporesponsiveness to iron and Erythropoietin Stimulating Agent (ESA) therapy. The complexity inherent in inflammation-induced elevated serum ferritin and hepcidin levels poses complications when setting predictive cut-off values for these biomarkers of iron deficiency anemia in CKD [10]. Hence, inflammatory confounders are a contentious issue attracting debate on consensus values for international guidelines on biomarkers of ferritin and hepcidin levels, as well as for iron and ESA dosage and routes of administration. Untreated anemia triggers several debilitating symptoms, such as lethargy, muscle fatigue, and deterioration of renal function. These culminate consequently in high prevalence of cardiovascular diseases, such as left ventricular hypertrophy and heart failure, which constitute the main causes of death in patients with CKD [11,12]. 

## 2. Anemia of Chronic Kidney Disease (ACKD)

**Figure 1:**
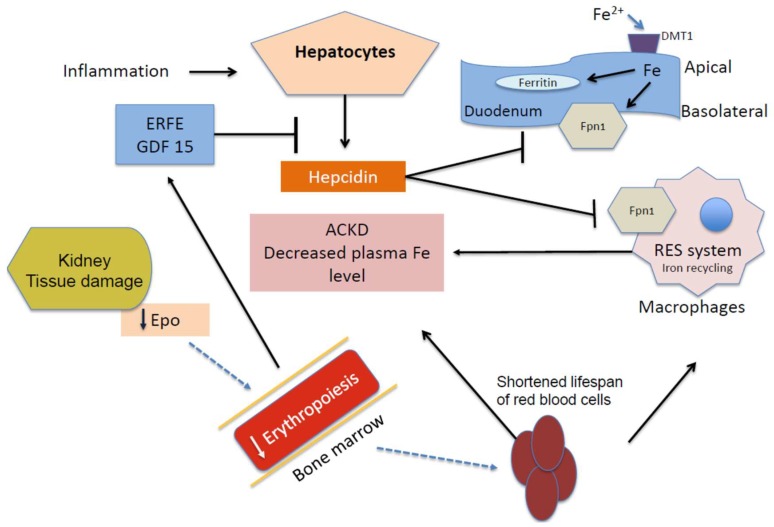
Iron metabolism in anemia of kidney disease. Hepcidin increases during inflammatory conditions and its clearance decreases in dysfunctional kidney cells. Fpn1 is degraded by hepcidin and, as a result, iron transport in the basolateral membrane of enterocytes reduces, as well as the mobilization of iron in macrophages, resulting in lower plasma levels of iron. The hepcidin level decreases during ineffective erythropoiesis and anemia by the actions of erythroid regulators erythroferrone (ERFE) and growth differentiation factor 15 (GDF15). EPO: Erythropoietin; Fpn1: Ferroportin; DMT1: Divalent Metal Transporter; and NTBI: Non-Transferrin Bound Iron.

Anemia is a common complication in CKD that increases in prevalence as the disease progresses [1]. Anemia is defined as Hb concentrations <13.0 g/dL in men and <12.0 g/dL in women [13]. Suboptimal levels of Hb and hematocrit in CKD patients are associated with declining survival rate [14,15]. This was evident in a population study that reported anemia as a critical factor in the development of cardiovascular disease (CVD) in CKD patients [16]. Consequently, CVDs such as heart failure and stroke have been implicated as major causes of mortality in CKD patients [11,12,17]. Anemia of CKD could also be due to multifactorial causes (Figure 1). Dysfunctional platelets, the shortening life span of red blood cells, iron deficiency, and inflammation are some of the factors that can trigger the onset of anemia [18]. The primary cause of anemia, however, is iron deficiency which may, in turn, be caused by low iron intake, low iron absorption, or disruption of body iron regulation. The damage that is caused to the kidney induces rapid activation of the immune system, and the inflammatory response, which stimulates IL-6 signal enhancement of hepcidin in the liver [19,20]. Inflammation inhibits erythropoiesis, affects erythropoietin (EPO) hyporesponsiveness [21], and reduces systemic circulation of iron levels by the production of hepcidin [22,23]. This response cascade indirectly contributes to the development of iron deficiency anemia (IDA) [24]. Excess hepcidin causes reduced circulation of iron in the plasma by a mechanism that involves the degradation of ferroportin, the iron efflux protein. Subsequently, iron release into the circulation from enterocytes and macrophages decreases [25,26] as shown in Figure 1. Levels of EPO decrease as a result of kidney damage and this culminates in lower erythroid cell production in the bone marrow. Bleeding during CKD causes loss of red blood cells leading to the development of anemia during CKD. Thus, the etiology of ACKD is a spectrum that involves both absolute and functional iron deficiency. The latter is compounded by an interplay of inflammation, tissue iron sequestration, and a hyporesponsiveness to ESA therapy [27]. Hypoxia-Inducible Factors (HIFs) that are secreted in the kidney during hypoxia can induce EPO production, and provide alternative therapy for (ACKD) [28]. Antibodies against hepcidin have been proposed as alternative approaches to increase iron absorption and iron efflux from the tissues [29].

## 3. Treatment of ACKD

Reduced production of red blood cell is proposed as the main cause of anemia in CKD patients. This arises from damage to the peritubular cells of the kidney, which produce erythropoietin (EPO), an essential hormone in erythropoiesis [30,31]. Erythropoietin induces the production and proliferation of erythrocytes; hence, a disruption in normal EPO levels causes anemia [32], as shown in Figure 1. Thus, the treatment for ACKD implies the administration of Erythropoiesis Stimulating Agents (ESAs). Recombinant human EPO (rHuEPO) is medically prescribed and several guidelines for promoting its efficacy in alleviating Hb levels have been reported [13,32,33].

The administration of rHuEPO is effective for correcting anemia and increasing hematocrit and reticulocyte count, although a concomitant increase in hypertension among the patients has been reported [34,35]. Currently, a Hb target range of 11.0 to 12.0 g/dL is recommended [36] because full normalization (Hb > 13 g/dL), according to Correction of Hemoglobin and Outcomes in Renal Insufficiency (CHOIR), is not prescribed due to increased cardiovascular events [37]. Additionally, in a post-hoc analysis of the CHOIR trial [38], increased mortality was found to be significantly associated with both the inability to achieve the Hb target and the use of high ESA doses. This was confirmed by a meta-analysis of 24 randomized controlled trials (RCTs), in which higher Hb targets resulted in increased hypertension risk (RR = 1.40, 95% confidence interval (CI) 1.11–1.75), stroke (RR = 1.73; 95% CI 1.31–2.29) and hospitalization (RR = 1.07, 95% CI 1.01–1.14) [6]. It was reported that rHuEPO induces hypo-responsiveness at high doses [39], and causes a greater risk of death due to the oxidative stress that exacerbates cardiovascular risk [40]. Recombinant HuEPO treatment is furthermore associated with increased blood pressure and blood clotting [41,42]. rHuEPO therapy is associated with iron deficiency as iron stores are largely transferred from the bone marrow to the erythroid progenitor cells due to enhanced erythropoiesis. Iron deficiency is observed in most hemodialysis patients arising from recurrent chronic blood losses. Thus, iron supplementation is often required to optimise or complement rHuEPO administration in the treatment of anemia in patients with CKD [18]. Consequently, if iron therapy is used alongside ESAs, significant increases in Hb levels and response are observed without the need to increase ESA dosage [43]. Thus, in practice, Kidney Disease Improving Global Outcomes (KDIGO) guidelines recommended a trial of intravenous iron to treat anemia in CKD, irrespective of ESA treatment [44]. In advanced stages of kidney disease, intravenous (IV) iron in combination with EPO therapy is currently the most effective treatment [44,45]. In light of this, the use of high intravenous iron doses was adopted in the U.S., despite the concerns raised by nephrologists regarding the resultant iron overload [46]. Supporting evidence from an analysis of 32,435 hemodialysis patients showed increased mortality (hazard ratio (HR) = 1.13, 95% CI 1.00–1.27) and hospitalization (HR = 1.12, 95% CI 1.07–1.18) in those receiving above 300 mg/month of intravenous iron compared to other patients receiving only 100 mg/month [47].

Although IV iron and rHuEPO led to improvement of hematological profiles, the risk of toxicity caused by excess iron predisposes patients to oxidative stress, inflammation, and pathogenic consequences [48,49]. Allergic reactions and anaphylactic shock, as well as oxidative stress that is related to cardiovascular complications and tissue injury, have been reported during the administration of IV iron supplementation [46,48,50]. The basis and mechanisms of the oxidative stress are not completely understood; however, excess iron from IV administration could cause iron overload and increase the levels of ROS in patients [12,51]. Also, IV iron supplementation raises levels of malondialdehyde (MDA), a biomarker of lipid peroxidation [52]. 

Several iron compounds including iron isomaltoside, iron sucrose, iron dextran, iron gluconate, or ferric carboxymaltose [53] are used for the treatment of ACKD. Intravenous iron formulations are colloidal suspensions, composed of a core of iron (iron-oxyhydroxide/oxide) surrounded by a carbohydrate shell [54]. The iron formulation varies in core size, affinity of bound iron to carbohydrate excipient, and electrovalence of iron, all of which influence the reactivity of iron [55]. 

Exposure of CKD patients to high concentrations of iron supplementation thus poses a potential risk of ROS generation with concomitant damage to DNA, proteins, or lipids [56]. Iron supplementation in patients with ACKD can subsequently result in iron overload, characterized by a “spill over” into hepatocytes if non-transferrin bound iron (NTBI) is present. Clinically relevant concentrations of NTBI would be expected if the iron-carrying capacity of transferrin is saturated [57]. Recommendations for iron management in CKD patient care are currently conflicting and is an ongoing process because of limited research evidence. A number of randomized controlled trials (RCTs) and observational studies have produced varying results on the effectiveness and adverse effects of iron or ESA supplementation. Variability or confounders, mostly associated with study design, have been identified [58] including type, dosage, duration or route of iron administration, population size, and the inherent variability within the baseline hematological status of patients.

## 4. Iron, Oxidative Stress, and Anemia

Iron (Fe), when supplied in excess, leads to oxidative stress in the mitochondria. Iron molecules trigger the initiation of the Fenton reaction and promote the formation of ROS, such as O^.^_2_^−^, OH^.^, and H_2_O_2_, as depicted in the equation: Fe^2+^ + H_2_O_2_ → Fe^3+^ + OH^.^ + OH^−^ [59]. These reactive species bind to macromolecules, such as lipids, proteins, and nucleic acids, causing lipid peroxidation and oxidative modifications of proteins and DNA [60]. Peroxidation of membrane lipids results in loss of membrane fluidity, elasticity, and disordered cellular functioning. Protein oxidation causes fragmentation of amino acid residues leading to cross-linkage and loss of protein configuration and functions. Oxidative damage of DNA causes mutation in DNA bases. These aberrations play major roles in cell death, ageing, and in degenerative diseases [61]. Observations in clinical trials showed a significant increase in the levels of MDA, a key oxidative stress marker, after IV iron infusion [62], and this is correlated with markers of early atherosclerosis [63]. Patients with CKD have a reduced mitochondrial DNA copy number, reduced energy production, and higher levels of stress markers [64]. Cytochrome c oxidase, an enzyme of the oxidative chain, is reduced in patients suffering from CKD in the final stages [65,66]. In addition, there are increasing concerns regarding the risk of iron therapy in potentially exacerbating oxidative stress, inflammation, and adverse cardiovascular outcomes from excess iron deposition in this population [11]. Atherosclerosis caused by oxidative damage, and evidenced by increased circulating mononuclear superoxide production and vascular cell adhesion molecule-1 (VCAM-1) and triggered by NADPH oxidase (NOx) and NF-kB activation in CKD patients, is associated with IV iron administration [67,68,69,70]. Against this evidence, some studies on the role of iron in CKD pathogenesis showed contradictory results [71]. Iron deposition in the liver of both humans [72] and rats [73] did not develop into cirrhosis, possibly because of iron sequestration into innocuous ferritin L and H subunits. Another mechanism that was proposed as a process that prevents iron overload in tissues such as the liver is the secretion of iron-loaded ferritin [74], possibly by an iron-regulated exocytosis efflux process [75] into blood circulation. However, other evidence revealed that high doses of iron were associated with high mortality due to iron-induced oxidative stress [68,71]. 

CKD patients, apart from a dysregulated iron metabolism, often exhibit hyperphosphatemia, which is associated with vascular calcification [69,76]. Paradoxically, heme iron, rather than being a pro-oxidant, was found to prevent the calcification and osteoblastic differentiation of human aortic smooth muscle cells (HSMCs) [77,78]. Evidence in this study attributed the inhibition of calcification to the upregulation of ferritin in the cells, even in the presence of phosphate. Heme releases Fe, CO, and biliverdin when catabolized. Thereby, iron performs the dual role of chelating phosphate and inducing the transcription of ferritin [79], particularly H-ferritin that exhibits high ferroxidase activity. Although the molecular mechanisms of vascular calcification require further investigation, emerging evidence indicates that ferritin might also function as a transcriptional regulator of gene expression in osteoblastic differentiation and β-globin synthesis [80].

## 5. Cytoprotective Functions of Histidine against Iron Toxicity during the Treatment of ACKD

L-histidine, a conditionally essential amino acid in adults, was found at significantly lower levels in patients with kidney disease and uremia [81,82]. Histidine, when administered orally or intravenously co-supplemented with iron, showed a positive response as judged by anemia markers, increased levels of plasma iron and Hb during anemia [83,84,85]. 

The beneficial effect of histidine is partly mediated by its ability to promote net nitrogen synthesis [84], which prevents negative nitrogen balance and loss of protein in CKD patients. Combined supplementation of IV iron with histidine rather than IV iron alone was more effective in treating anemia in CKD patients [86]. Additional evidence supports the beneficial role of histidine supplementation in uremic and dialysis patients, as a slight increase in Hb levels was triggered. Low histidine levels were shown to be correlated with high mortality (HR = 1.55, 95% CI 1.02, 2.40, *p* < 0.05), even after adjustments for age, sex, cardiovascular disease, inflammation, diabetes mellitus, serum albumin, and amino acid supplementation [81]. Histidine administration was negatively correlated (*p* < 0.05) with levels of 8-OHdG, an oxidative stress biomarker [81]. Histidine is known as an efficient scavenger of ROS [87], and its antioxidant properties have been advocated in the prevention of iron toxicity. 

## 6. Antioxidant Function of Histidine against Oxidative Stress

The management of anemia of CKD presents a conundrum that arises from a role in the treatment of iron deficiency and in improvement of the resultant toxicity of excess iron. Endogenous and dietary antioxidants prevent, neutralize, and terminate chain reactions that produce ROS. 

Studies by Halliwell and Wade identified that histidine could serve as an antioxidant as well as a buffer, similar to albumin in the plasma [88,89]. Hence, histidine could function as a buffer regulating free metal ion concentration, thereby providing a safe temporary transport for divalent metals before they are metabolized. In cardiovascular studies, histidine afforded protection to the cardiovascular system because of its ability to scavenge singlet oxygen and hydroxyl radicals in isolated hearts tissues that are predisposed to oxidative stress [90]. The scavenging of singlet oxygen by histidine was found to be significantly higher than that of tryptophan or methionine [89]. Moreover, histidine is suggested to be protective against oxidative stress in a drug model, reflected in its storage stability [91]. Histidine has been shown to have an inhibitory effect on H_2_O_2_-induced IL-8 secretion in Caco-2 and HT-29 cells [92]. Lipid peroxidation was considerably inhibited when histidine was added to ferric iron in vitro [93], suggesting that histidine formed a complex with ferric iron and prevented the formation of ferrous iron and the Fenton reaction [94]. Histidine quenches and scavenges hydroxyl radicals and singlet oxygen [89]. The intracellular concentration of histidine is higher than found in plasma [89,95], resulting in different cellular responses in the protection against ROS. Histidine and its dipeptides have also been associated with increased expression of catalase and glutathione peroxidase antioxidant enzymes [96,97]. Although histidine exerts a positive effect on the amino acid pool within the cell, the deprivation of histidine could specifically induce a decrease in enzymatic antioxidant defenses [98]. The use of histidine as a scavenger increases the defense of cells against oxidative damage [99]. Histidine supplementation was shown to enhance the expression and the activities of catalase and glutathione peroxidase (GPX), in response to ethanol-induced liver damage in mice [100]. Consequently, the potential resides in histidine to enhance enzymatic antioxidant activity during oxidative stress and inflammatory conditions in the cell [100,101,102].

A study of the cytoprotective effect of histidine on the nervous system was related to the activity of histidine in the transport of glutamine into mitochondria during edema and inflammatory conditions [97]. As L-histidine readily traverses the blood-brain barrier, supplementations with this amino acid were shown to increase both total nitric oxide synthases (NOS) and total antioxidant capacity, conferring protection against oxidative stress and encephalopathy in rats [103]. Moreover, histidine was reported to be a scavenger of the hydroxyl radical in a study conducted on rabbits [104]. The scavenging of singlet oxygen by histidine was further confirmed in rats [105]. Histidine is, therefore, regarded as an efficient scavenger of the both hydroxyl radical and singlet oxygen based on its antioxidant abilities [87], as well as possessing the capacity to chelate divalent metals such as iron [51,54,63]. 

**Figure 2:**
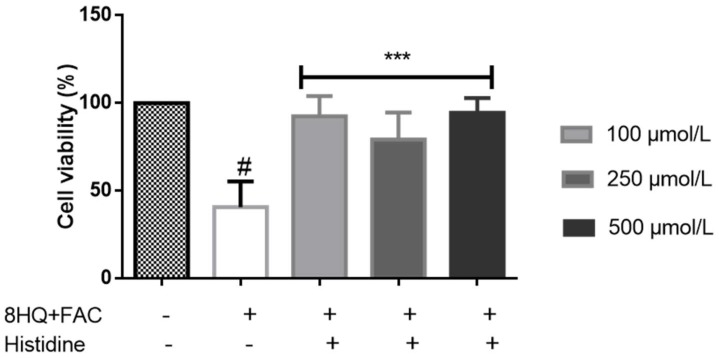
Protective effect of histidine against iron-induced stress in HEK-293 cells. Cells were treated with histidine (100–500 µM) overnight and subjected to 20 µM 8-hydroxyquinoline (8HQ) and 50 µM ferric ammonium citrate (FAC) for two hours, after which cell viability was performed using 3-(4,5-dimethylthiazol-2-yl)-2,5-diphenyltetrazolium bromide (MTT) assay. *** *p* < 0.001 between 8HQ + FAC and the treatments, # *p* < 0.001 between the control and iron treatment.

Histidine was shown to protect against iron-induced oxidative stress in human embryonic kidney (HEK-293) cells (Figure 2). Cells were pre-treated with histidine at 100, 250, and 500 µM concentrations overnight, and then subjected to iron challenge with 20 µM of 8-hydroquinoline (8-HQ) and 50 µM of ferric ammonium citrate (FAC) for two hours. Histidine significantly protected cell viability in cultured HEK-293 cells at all histidine concentrations [106]. This finding confirms earlier results from the literature on the lowering of inflammation and oxidative stress by histidine in other cell culture models [92]. The molecular mechanisms by which histidine exerts the protective function against iron and oxidative stress require further investigation.

## 7. Metal Chelation Capacity of Histidine 

**Figure 3:**
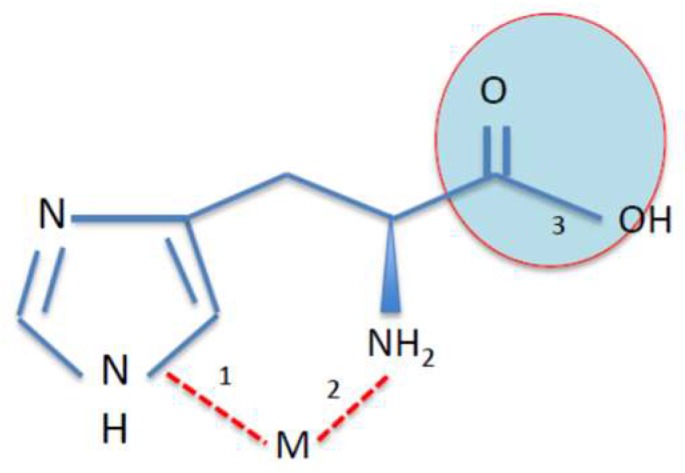
Structure of the histidine molecule showing the imidazole ring, amino, carboxyl groups, and metal ion (M) binding sites.

**Figure 4:**
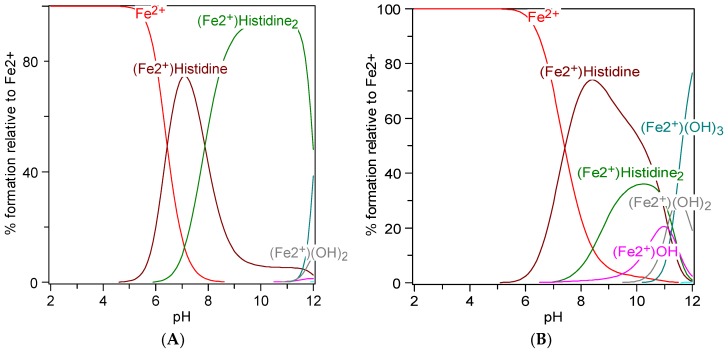
Speciation plots of histidine and ferrous (Fe^2+^, Fe (II)) iron. Speciation plots with parameters of 50 µM Fe (II) and (**A**) 1 mM histidine or (**B**) 100 µM histidine.

Free histidine is an amino acid that is present in cells of the brain, skeletal muscle, and liver [100]. It has an imidazole ring that, in combination with the amino group, facilitates binding and chelation of other compounds. Histidine is also a constituent of some dipeptides such as carnosine, anserine and a precursor of the neurotransmitter histamine [89,96,107,108]. Notably, the iron-chelating property of carnosine has been ascribed to the imidazole ring of histidine [96,102,108]. Histidine is a proximal ligand of heme iron in the Hb molecule [109]. In equimolar solutions, divalent metals can interact with histidine via the amino, imidazole, or carboxyl moiety (Figure 3). This is probably highly specific and selective amongst different divalent metal ions. The formation and stability of most histidine-divalent metal complexes are favored by slightly acidic pH (pH < 5). The histidine molecule thus forms stable strong bonds with iron ions, displaying a bidentate and protonated activity on the primary amino-group at pH 5. Tridentate metal chelate can also form with histidine via the imidazole ring [110,111,112]. Histidine forms stable bidentate complexes in aqueous systems with Cu^2+^, Fe^2+^, and Ni^2+^ [112,113]. Incidentally, complexes of histidine with Zn^2+^, Cu^2+^, Ni^2+^, or Co^2+^ have higher stability constants than with Fe^2+^, and the binding stability is dependent on the binding moiety, temperature, and pH of the solution [112]. Histidine-divalent metal (Cu^2+^, Zn^2+^, and Ni^2+^) complexes, in aqueous solutions, form with the imidazole and amine group at pH 6 [111]. The iron chelating property of histidine is therefore dependent on pH and the nature of the three possible interacting bonds with ligands (Figure 3). The pKa values of histidine in proteins range between pH 6 and pH 8. Consequently, side chains of histidine contribute to the buffering potentials of proteins such as Hb at the physiological pH of blood. A simulation of the titration curve of histidine reacting with ferrous iron, presented in Figure 4, shows that histidine at 1 mM forms a complex with iron at neutral pH; however, when the concentration of histidine is lowered 10 fold, the formation of the complex decreases and peaks at pH 8. This speciation plot shows that, at neutral pH, histidine, at high concentrations, can bind iron with high affinity; however, this reaction is not at the physiological concentration of histidine in tissues or plasma. 

In vitro studies demonstrated that histidine is the most effective hydroxyl radical scavenger out of the several amino acids that were investigated [114]. The scavenging ability of histidine seems to involve a chelating mechanism that interferes with the redox reaction of metal ions producing hydroxyl radicals [89]. Nair et al. [115] reported that histidine displays a strong binding affinity to Fe^3+^ ions, thereby reducing the amount of ROS generated via the Fenton reaction, and protecting cells from damage due to iron overload [87]. In addition, histidine can interact directly with singlet oxygen through its imidazole ring [89]. Some other studies have shown, in contrast, that histidine may function as a pro-oxidant. Tachon reported that the enhancing effect of histidine on DNA degradation by ferric ions is dependent on the chelator/metal ratio, and is likely mediated by an oxidant such as ferrous-dioxygen-ferric chelate complex or a chelate-ferryl ion [116]. Evidence from studies to support the protective effect of histidine against oxidative stress remains both fragmentary and contradictory. This discrepancy is perhaps due to differences in experimental methodology and design; for example, the types of established cell lines, and histidine dosage employed in the studies. 

The chelating property of histidine has been correlated with some physiological functions such as the capacity to increase iron uptake [117], as well as its ability to protect against ROS in the nervous system [92]. Histidine has the capacity to bind divalent metal ions (including those of copper, zinc, and iron) and enhance iron absorption [117,118]. The ligand formed by histidine possibly prevents iron precipitation, enhancing iron absorption in the intestine [118,119]. Histidine-containing peptides chelated iron for enhanced iron absorption in the segments of rat intestine [120]. This is, however, in contrast to an earlier finding in a human study in which histidine did not enhance iron absorption [121]. The ratio of histidine content in different peptides has been contradictorily reported regarding either its inorganic iron absorption or enhancing function [117].

Histidine has been associated with the capacity to chelate iron and enhance iron solubility in the duodenum, as ferrous iron is poorly soluble at neutral pH. By binding to histidine and other chelating compounds, the uptake of the metal increases [119]. The role of histidine in binding iron and increasing its uptake is debatable; the positive evidence suggested that the co-supplementation of histidine doses of about 100 mM could increase iron uptake in rats, and in combination with ascorbic acid, this could doubly enhance the positive effect of ascorbic acid [122]. This was also observed in cultured Caco-2 cells [123]. However, another study on intestinal cells and on humans did not report such increases of iron uptake upon histidine supplementation [121,124]. In food extracts, histidine has been found to increase inorganic iron solubility; however, these results arose from vegetables and meat extracts wherein the efficacy of histidine could have been confounded by the presence of other amino acids and peptides [117,125,126]. Histidine undoubtedly has the capacity to bind iron; however, as this reaction is pH-dependent, histidine might serve the important function of maintaining iron in soluble form to enhance iron bioavailability. The presence of other compounds or stronger metal chelators could influence the stability of the histidine-iron complex. 

## 8. Anti-Inflammatory Potential of Histidine 

As inflammation promotes iron disorders in anemia of chronic kidney disease, factors that increase or inhibit inflammatory signals are important in the manifestation of the disease symptoms. Inflammatory conditions or responses lead to the secretion of different cytokines, such as interleukin 6 (IL-6), interleukin 8 (IL-8), interleukin-1beta (IL-1b), as well as tumor necrosis factor-alpha (TNF-α) [92]. The physiological effects of histidine as an antioxidant have been associated with the inhibition of the secretion of some of these pro-inflammatory biomarkers, particularly during chronic diseases [89].

Liu et al. [100] reported that mice fed with histidine (at 0.5, 1, or 2 g/L) after the induction of ethanol hepatotoxicity had lower levels of the inflammation markers IL-6, c-reactive protein (CRP), and TNF-a. Histidine suppressed the accumulation of IL-6 and TNF-α mRNA in a dose-dependent manner. This indicates that histidine is not only a scavenger of ROS but could directly affect the regulation of pro-inflammatory cytokines [100]. A similar effect was found in cultured cells, in which histidine protected Caco-2 cells in a dose-dependent manner against hydrogen peroxide-induced damage [89]. Histidine, apart from reducing morphological damage in these cells, suppressed the secretion of IL-8 as well. This outcome is presumably due to the inhibition of signals for TNF-α and NF-kβ to activate transcriptional regulation of IL-8 [92]. Down-regulation of IL-6 and TNF-α by histidine has been reported in a diabetic mouse model [127]. The inhibition of pro-inflammatory cytokines by histidine is linked to its potential to increase the amino acid pool and the antioxidant status of the cells [107]. These changes contribute to the attenuation of ROS in tissues and organs. Histidine was reported to alleviate clinical symptoms and protect mice from intestinal, nervous, and cardiovascular damage [89]. These effects are possibly associated with the anti-inflammatory properties of histidine. Consequently, histidine administration could be an option for reducing and preventing oxidative stress in inflammatory conditions in chronic diseases. Dietary supplementation with histidine could be protective against oxidative damage, arising from the scavenging activity and anti-inflammatory effects of this amino acid in cells and tissues [127]. 

Arginine is another amino acid that exhibits antioxidant properties as a substrate in nitric oxide (NO) biosynthesis and was shown to be beneficial to kidney functions in both in vivo and in vitro models [128,129]. As well as other histidine dipeptides, carnosine also scavenges ROS and was reported to be beneficial to CKD patients [130]. 

## 9. Conclusions 

Anemia of chronic kidney disease is a worldwide public health problem, with a constantly-increasing trend in both developed and developing countries. Iron supplementation treatment of ACKD leads to oxidative damage that is associated with iron overload and pro-inflammatory conditions. Co-supplementation with histidine, could have an ameliorating effect against oxidative damage in chronic kidney disease. Histidine can protect against iron overload and oxidative stress caused by CKD, as demonstrated with the Fenton substrate in the HK-2 cell model. The molecular mechanisms of the cytoprotection conferred by histidine are based on its capacity and potential as a metal chelator as well as its ability to scavenge oxygen radicals. Histidine can also function intracellularly to induce enzymatic antioxidant and anti-inflammatory activities in tissues and organs in different disorders [113,114]. The imidazole ring of histidine, as well as binding singlet oxygen molecules, can bind divalent metal ions; however, the effects of pH and histidine concentration that maintains iron in solution have only been clearly demonstrated in cultured cells and in animal models. It remains for these effects to be demonstrated in human subjects. The iron quenching capacity appears to be related to the reactive ability of the imidazole ring of histidine in extracellular solutions. No clear evidence currently exists for the localization of histidine-iron bound complexes or chelates intracellularly.

Further studies are required to clearly delineate the molecular mechanisms by which histidine attenuates oxidative stress damage attendant upon iron toxicity in kidney cells and specific models during the treatment of ACKD.
